# A natural probioenzyme strategy as an effective alternative to antibiotics against multidrug-resistant *Klebsiella pneumoniae* in broilers: Benefits for growth, immunity, and organ health

**DOI:** 10.1371/journal.pone.0343093

**Published:** 2026-03-27

**Authors:** Atef A. Salim, Nehal A. Naena, Mofid Y. Gouda, Safaa M. Shabana, Amr I. Zaineldin

**Affiliations:** 1 Poultry diseases, Kafrelsheikh branch, Animal Health Research Institute (AHRI), Agricultural Research Center (ARC), Giza, Egypt; 2 Bacteriology Department, Kafrelsheikh branch, Animal Health Research Institute (AHRI), Agriculture Research Centre (ARC), Giza, Egypt; 3 Pathology Department, Kafrelsheikh branch, Animal Health Research Institute AHRI, Agriculture Research Centre (ARC), Giza, Egypt; 4 Animal Nutrition, Aquaculture Department, Faculty of Aquatic and Fisheries Sciences, Kafrelsheikh University, Kafrelsheikh, Egypt; 5 Unit of Biochemistry, Nutritional Deficiency Diseases and Toxicology, Kafrelsheikh branch, Animal Health Research Institute (AHRI), Agriculture Research Center (ARC), Giza, Egypt; Tanta University Faculty of Agriculture, EGYPT

## Abstract

The rise of multidrug-resistant *Klebsiella pneumoniae* poses a serious threat to poultry health and food safety, necessitating sustainable alternatives to antibiotics. Our investigation began with an epidemiological survey of broiler samples, which detected *K. pneumoniae* in 8.88% (8/90) of cases. Among these isolates, over 60% were multidrug-resistant. Antimicrobial susceptibility testing showed bacterial resistance to penicillin G (100%) but complete sensitivity to colistin sulphate (100%). Molecular analysis further revealed a high prevalence of key resistance genes (*tet A* [87.5%] and *bla*_TEM_ [100%]) and virulence factors (*fim H* [100%] and *tra T* [62.5%]), while all isolates were confirmed by amplification of the conserved 16S-23S ITS region. We then evaluated a novel probioenzyme formulation as a dietary intervention in a 35-day broiler trial. Birds challenged with *K. pneumoniae* were supplemented with either probioenzyme (1 g/kg feed) or treated with colistin sulfate (antibiotic control). Probioenzyme supplementation resulted in growth performance enhancement (final body weight, weight gain, specific growth rate) comparable or superior to, the antibiotic-treated group (*P < 0.05*). The probioenzyme in KPT restored FCR to levels matching the unchallenged (CT) and antibiotic-treated groups (KAT). Histopathological analysis showed complete restoration of intestinal villus architecture, pulmonary integrity, and hepatic cytoprotection in the probioenzyme group, matching antibiotic efficacy. Furthermore, probioenzyme enhanced systemic antioxidant capacity (catalase, superoxide dismutase, total antioxidant capacity) and innate immune response (lysozyme activity) by day 35 (*P < 0.05*). These findings demonstrate that dietary probioenzyme is a viable alternative to antibiotics, effectively mitigating *K. pneumoniae* infection while improving growth, organ health, and immune competence without contributing to antimicrobial resistance. This strategy supports sustainable poultry production and aligns with global efforts to reduce antibiotic reliance.

## Introduction

Despite significant progress in chicken housing and nutrition, bacterial diseases remain a major source of economic losses in poultry farming. One contributing factor is the strict regulations in modern farming practices, which limit the time newly hatched chicks spend near older birds. This reduces their exposure to beneficial (probiotic) bacteria from the gastrointestinal tracts (GIT) of mature hens, which are crucial for early immune development [1,2]. As a result, newly hatched chicks are particularly vulnerable to infections, especially within the first 24 hours after hatching.

Among emerging pathogens, *Klebsiella pneumoniae* represents a particularly concerning challenge. As a Gram-negative, zoonotic bacterium, it not only causes respiratory and gastrointestinal infections in poultry [3] but also serves as a reservoir for multidrug resistance (MDR) genes. As the prevalence of antibiotic-resistant *K. pneumoniae* strains increases, treating infections caused by this bacteria has become increasingly challenging, highlighting the need for alternative strategies to combat such challenges in poultry health management [4].

The growing resistance of bacteria to antibiotics is a well-documented concern, compounded by factors such as β-lactamase enzymes, efflux pump activity, and genetic mutations that disrupt porin function or alter penicillin-binding protein (PBP) expression [5].

Nowadays, the worldwide pattern in animal production is towards an increase in the application of non-antibiotic approaches that can provide comparable benefits. This is due to the fact that the widespread use of antibiotics over the last 50 years has led to the emergence of resistant bacteria and drug residues in animal products [6–8].

Presently, the poultry industry uses probiotics and prebiotics to enhance gut health concerns. These beneficial feed additives can help exclude pathogens, and activate the immune response, resulting in better animal performance and lower disease susceptibility [9,10]. As the poultry industry increasingly looks for alternatives to antibiotic, the adoption of probiotics and other feed additive that improve gut health and immunity has become a crucial element in achieving sustainable and efficient poultry production [11].

Probiotics are widely recognized for their multifaceted biological activities, including antibacterial, antioxidant, antifungal, and antihepatotoxic properties, as well as their cytotoxic effects [12,13]. Numerous studies have explored the antibacterial potential of probiotics, with *in vitro* research demonstrating their efficacy against a broad spectrum of Gram-positive and Gram-negative bacteria [14–17].

Based on this, we hypothesized that dietary supplementation with a multi-strain probioenzyme formulation would be as effective as therapeutic antibiotic treatment in mitigating the negative impacts of *K. pneumoniae* infection in broilers. Specifically, we postulated that probioenzyme would enhance growth performance, restore gut and organ morphology, and improve systemic antioxidant status and immune response to a level comparable to antibiotic-treated birds. Therefore, this study was designed to evaluate the efficacy of the probioenzyme formulation as a therapeutic intervention, directly compared to a last-resort antibiotic (colistin sulfate), in broilers actively infected with a characterized MDR *K. pneumoniae* strain. The findings will provide insights into the potential of probioenzyme as a dietary intervention to enhance broiler health, productivity, and disease resistance, as well as explore its potential as a more effective alternative to synthetic antibiotics in broilers infected with *Klebsiella pneumoniae*.

## Materials and methods

### Klebsiella pneumoniae isolation

#### Sample collection.

A total of 90 pooled samples, including lung, trachea, liver, and cloacal swabs, were collected from broiler farms in Kafr El-Sheikh Governorate, Egypt, where birds exhibited respiratory symptoms. These samples were aseptically transported in an icebox to the laboratory at Animal Health Research Institute- Kafr El-Sheikh branch, for bacteriological analysis.

#### Necropsy procedures.

Following standard necropsy protocols, all birds underwent thorough clinical and postmortem examinations. Using sterile techniques, researchers collected 100 biological specimens from four anatomical sites: pulmonary tissue, splenic tissue, hepatic tissue, and cloacal swabs for subsequent bacteriological analysis.

#### Bacteriological isolation and Identification of *K. pneumoniae.*

Samples from lung, trachea, liver, and cloacal swabs were cultured in nutrient broth and incubated aerobically at 37°C for 24 hours. Following primary enrichment, broth cultures were streaked onto MacConkey agar (Oxoid, UK) and incubated at 37°C for 24–48 hours. Suspected *K. pneumoniae* colonies (large, mucoid, pink) were subcultured onto EMB agar for purification. Identification was performed through a combination of colony morphology (mucoid pink/purple colonies on EMB), Gram staining (Gram-negative rods), and comprehensive biochemical testing, including positive reactions for catalase, urease, citrate utilization, lactose fermentation, and Voges-Proskauer tests, along with negative results for oxidase, indole production, and H2S formation. The identification protocol followed standard microbiological procedures [18–20]*,* with biochemical profiling serving as confirmatory for *K. pneumoniae*. Moreover, the identified isolates were further confirmed through amplification with species-specific 16S rRNA primers.

### Antibiotic susceptibility test (AST)

The Kirby-Bauer disk diffusion method was used to identify the susceptibility of *K.pneumoniae* isolates to a group of antibiotics [21]. Nine antibiotic discs from different classes were tested: Penicillin G (P, 10 μg), Amoxicillin (AMX, 25 μg), Cefotaxime (CTX, 30 μg), Gentamycin (CN, 10 μg), Streptomycin (S, 25 μg), Ciprofloxacin (CIP, 5 μg), Nalidixic Acid (NA, 30 μg), Tetracycline (TE, 30 μg), and Colistin/Sulphate (CTS, 10 μg). Antimicrobial susceptibility testing was performed using Mueller-Hinton agar (Oxoid, UK). Following a 24-hour incubation period at 37°C, an isolated colony was suspended in sterile 0.9% saline solution. The bacterial suspension was standardized to a 0.5 McFarland turbidity standard (approximately 1.5 × 10⁸ CFU/mL) and uniformly spread onto Mueller-Hinton agar plates using a sterile cotton swab. Antibiotic discs were then aseptically placed on the inoculated plates and incubated at 37°C for 18–24 hours. The procedure adhered to the standardized protocols established by the Clinical Laboratory Standards Institute (CLSI) [22]. Following incubation, the diameters of inhibition zones were measured, and bacterial isolates were classified as sensitive (S), intermediate (I), or resistant (R) based on established breakpoints.

#### MAR index determination methodology.

To quantify multiple antibiotic resistance, the MAR index was computed by taking the ratio of resistant antibiotics (numerator) to the total antibiotics tested (denominator). An index value above 0.2 served as a critical threshold, signifying that the bacterial isolates likely came from environments with substantial antibiotic pressure [23]. The classification of multidrug-resistant (MDR), and the multiple antibiotic resistance (MAR) isolates were performed following the established criteria by [24].

### Molecular verification and resistance gene PCR analysis

#### Genomic DNA extraction.

DNA was extracted from the isolated samples using the QIAamp DNA Mini Kit (Qiagen, Germany, GmbH) according to the manufacturer’s protocol. Briefly, 200 µl of sample suspension was incubated with 10 µl of proteinase K and 200 µl of lysis buffer at 56°C for 10 minutes. After incubation, 200 µl of 100% ethanol was added to the lysate. The sample was then washed and centrifuged as per the manufacturer’s instructions. Nucleic acids were eluted using 100 µl of elution buffer.

Isolates were screened by PCR amplification using specific primers for the detection of the following genes: Tetracycline resistance gene: *tetA* (efflux-mediated tetracycline resistance); ESBL-encoding genes: *bla*_*TEM*_ (common extended-spectrum β-lactamase genes); *fimH* (the adhesive subunit of type 1 fimbriae); *traT* (outer membrane protein-coding gene); *K. pneumoniae 16S-23S ITS* (The 16S-23S rRNA gene internal transcribed spacer (ITS) regions of *Klebsiella*).

#### Oligonucleotide primer.

Primers were obtained from Metabion (Germany) and are listed in Table (1). PCR amplifications were performed in a 25 µl volume containing 12.5 µl of EmeraldAmp Max PCR Master Mix (Takara, Japan), 1 µl of each primer (20 pmol concentration), 5.5 µl of water, and 5 µl of DNA template. Amplification was carried out in an Applied Biosystem 2720 thermal cycler.

#### Assessment of PCR-generated DNA fragments.

PCR products were separated by electrophoresis on a 1.5% agarose gel (Applichem, Germany, GmbH) in 1x TBE buffer at room temperature using a voltage gradient of 5V/cm. For gel analysis, 20 µl of each product was loaded into the gel slots. A Generuler 100 bp ladder (Fermentas, Germany) and a GelPilot 100 bp ladder (Qiagen, GmbH, Germany) were used as size markers. Gels were visualized using a gel documentation system (Alpha Innotech, Biometra), and data were analyzed using computer software.

### In vivo experimental set-up

#### Ethical approval.

The experimental procedures and all methods followed the relevant guidelines and regulations established by the Research Ethics Committee of the Animal Health Research Institute-Agriculture Research Center (AHRI-ARC, Approval Number (**142429**)) in compliance with European Union Directive 2010/63/EU. All experimental procedures adhered to institutional guidelines and ARRIVE reporting standards (https://arriveguidelines.org). Research was conducted in controlled Pen facilities at the Animal Health Research Institute, Kafrelsheikh, Egypt, requiring no special access permissions. No human participants or protected species were involved in this study.

#### Animals and experimental design.

Experimental groups and feeding: The study employed a completely randomized design with 155 one-day-old male-Cobb broiler chicks (white-feathered broilers), exhibiting comparable initial body weights (56 ± 1 g). Initial bacteriological screening of five randomly selected chicks confirmed the absence of Klebsiella spp. contamination prior to trial initiation. The remaining 150 birds were systematically allocated across 15 cages (10 birds per cage), with equal distribution into five treatment groups (3 cages per group, totaling 30 birds per experimental condition). To prevent cross-contamination, birds in the uninfected control group were kept separate from those in the infected groups. All broilers were provided with a formulated mash feed diet ad libitum, following the recommendations of the Cobb Broiler Management Standards (Cobb Vantress). The diet was divided into three phases (Table 6): starter feed (23% CP for the first 13 days), grower feed (21% CP for the next 13 days), and finisher feed (19% CP for the final 9 days).

During the 35-day study, birds were divided into five treatment groups: (**1**) unchallenged control (**CT**) fed standard diets; (**2**) *Klebsiella pneumoniae*-challenged (**KT**) receiving standard feed; (**3**) challenged and antibiotic-treated (**KAT**); (**4**) probioenzyme-supplemented (**PT**, 1 g/kg feed); and (**5**) challenged probioenzyme group (**KPT**). All groups followed a three-phase feeding regimen: starter (days 1–13), grower (days 14–26), and finisher (days 27–35) diets, with the bacterial challenge administered according to experimental design. The *K. pneumoniae* strain that was previously isolated and identified biochemically and by molecular identification was sub-cultured in nutrient broth at 37^o^C for 24 hrs then centrifuged at 3000 r.p.m for 10 min. Bacterial pellets were then suspended in sterile physiological buffer saline and adjusted using MacFarland 0.5 tube to contain 1 × 10⁸ cfu/ml. The challenge dose was selected based on a preliminary pilot study to induce consistent clinical signs and pathological lesions without excessive mortality, ensuring a robust model for evaluating interventions. On day 10 post-hatch, groups KT, KAT, and KPT were challenged with 1 mL fresh *K. pneumoniae* culture (24-hr broth, 1 × 10⁸ CFU/mL) via oral gavage. KT group subsequently received therapeutic colistin sulfate (1 g/L drinking water) for five consecutive days beginning 48 hours post-infection. The antibiotic treatment (days 12–16 post-hatch) was completed six days before the day-22 growth measurement. Chicks were monitored daily for clinical manifestations following infection. Diseased or moribund birds underwent immediate necropsy with concurrent bacteriological analysis to confirm pathogen presence. Mortalities were recorded daily from the beginning of the experiment.

Probioenzyme: The supplementation dose (1 g/kg feed) was selected based on the manufacturer’s recommendation, which is derived from previous in-house efficacy trials and aligns with common application rates for similar probiotic-enzyme products in poultry nutrition. Probioenzyme-used was a commercial product (Nutrbact^®^, Vetcova Co, Egypt), a synergistic commercial blend of probiotics, prebiotics, and exogenous enzymes, that contained probiotic mixture (*Aspergillus Oryzae, B.subtilis, lactobacillus acidophilus, lactobacillus plantarum, bifidobacterium bifidum, Enterococcus faecium* with a concentration of 20x10^9^, colony forming unit (CFU) per gram), prebiotic mixture (mannooligosaccharide, 2.5 gm and beta glucan, 5 gm/kg) and enzyme mixture (xylanase, lipase, alpha amylase 5000 IU/kg, phytase 10000 IU/kg, cellulose 13000 IU/kg and protease 16000 IU/kg),Produced by VETCOVA for veterinary products and feed additives, batch no. VETCOVA Egypt, Batch no.2221004 and expiry date 8/2025.

Antibiotic (colistin): Contain colistin sulphate 16.66 gm, Produced by medical professions for veterinary products and feed additives (MUVCO), and Batch no.1904122 and expiry date 4/2026.

*Klebsiella* isolation from the cecum was conducted on broilers at 11, 14, 19, 21, 25, and 28 days of age. The spleen and bursa of Fabricius were excised from broilers at 14, 21, and 28 days of age, and serum samples were collected at 11, 14, 21, and 28 days of age. To minimize unnecessary culling, as many tests as possible were performed on the same bird.

### Growth performance

Growth performances of the birds were evaluated based on daily feed intake (FI) and changes in body weight throughout the trial. Bodyweight and feed intake were recorded weekly and the averages per cage calculated. All birds were weighed and the change in body weight calculated relevant to body weight recorded on day 1,10, 22, and 35. Average feed conversion ratio (FCR), specific growth rate (SGR), and feed conversion efficiency (FCE) were calculated from feed intake (FI) and body weight gain (WG). Mortalities were recorded throughout the study. The growth parameters were calculated accordingly using the following formulae:


Weight gain (g/bird) = [final body weight (FBW, g) − initial body weight (IBW, g)];

Feed conversion ratio (FCR) = total dry feed intake (FI, g) / Weight gain (WG, g);

Specific growth rate (SGR, %/day) = 100 × [ln FBW (g) − ln IBW (g)] / days;

Feed conversion efficiency= live weight gain (g)/dry feed intake (g).

### Gut, lung, and liver histomorphology

All broilers euthanized in accordance with the AVMA’s (American Veterinary Medical Association) Guidelines for the Euthanasia of Animals (2020). On days 18 and 35, broilers were euthanized by carbon dioxide asphyxiation [25],then slaughtered,and the abdomen was dissected, and tissue specimens from the intestine,lung, and liver of three birds per replicate (CT, KT,KAT,PT,and KPT) were randomly selected directly after euthanasia via controlled CO₂ inhalation. All samples were fixed in Bouin’s solution for 18 hours. Following fixation, the tissue samples were dehydrated using ethyl alcohol in increasing concentrations (from 70% to 100% alcohol), twice rinsed with xylene (1 and 2 h), and then embedded in paraffin. Sections of 5-µm thickness were gathered and stained with haematoxylin and eosin (H&E). From each tissue, two cross-sectional slices were made. The tissue sections were then stained with H&E before being examined using Sigma Scan Pro 5 software and a light microscope (Leica DM500) and camera (Leica EC3, Leica GmbH, Wetzlar, Germany) [26]. villi length, villi width, crypt depth and Musclaris thickness, were measured using ImageJ analysis software with magnifications of 100 × , 200 × , and 400 × .Ten measurements were taken for every tissue in accordance with an established technique [26].

### Serum antioxidants and lysozyme activity

On days 18 and 35, serum was collected from 9 birds (3 per cage) in each group from the brachial vein using a sterile syringe coated with heparin (100 U/mL). The serum was stored at − 80 °C. Superoxide dismutase (SOD), catalase (CAT), and Total antioxidant capacity (TAC) levels in serum were evaluated spectrophotometrically following the methods in previous studies [27,28], and [29], respectively). Serum lysozyme activity was evaluated according to a published method [30]. The lysozyme unit present in serum (μg/mL) was obtained by comparison with a standard curve produced using lyophilized hen egg white lysozyme [30,31].

### Statistical analysis

The collected data from all trial groups was statistically examined using statistical software (IBM SPSS for Windows, version 22, USA) in order to compare means with the control group. One-way analysis of variance (ANOVA) with Tukey testing was employed to assess differences between the means of the experimental groups. In order to show the significance between all groups, A *p* value < 0.05 was used.

## Results

### Clinical signs of the collected samples

**Fig 1:**
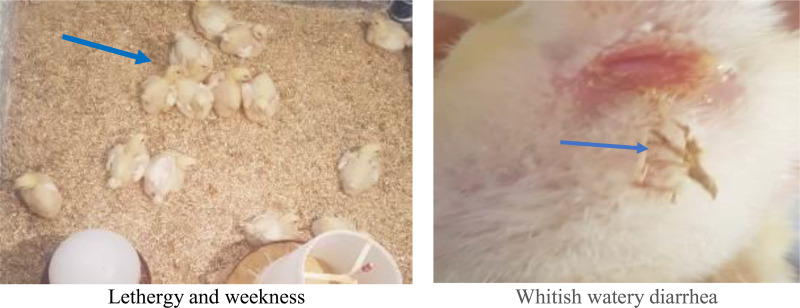
Clinical signs of birds infected with *Klebsiella pneumoniae.* **(A)** Lethergy and weekness. **(B)** Whitish watery diarrhea.

The birds of the collected samples were showing clinical signs of depression, significant body weight loss, ruffled feather, gasping, nasal mucoid discharge, diarrhea, and pasty vent plumage (Fig 1).

### Postmortem examinations the collected samples

**Fig 2:**
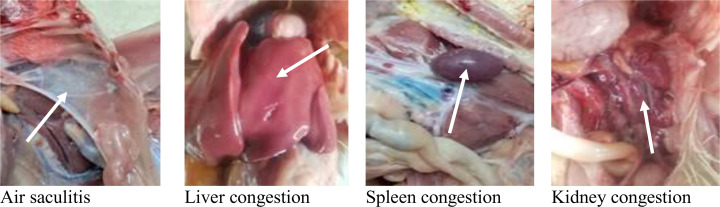
Postmortem findings in birds infected with *Klebsiella pneumoniae.* **(A)** Air saculitis. **(B)** Liver congestion. **(C)** Spleen congestion. **(D)** Kidney congestion.

Postmortem examination of the infected birds revealed air sacculitis (inflammation of the air sacs), severe congestion of the liver and spleen, and renal congestion with ureteral urate deposition (Fig 2).

### Isolation and identification of *K. pneumonia*e

**Fig 3:**
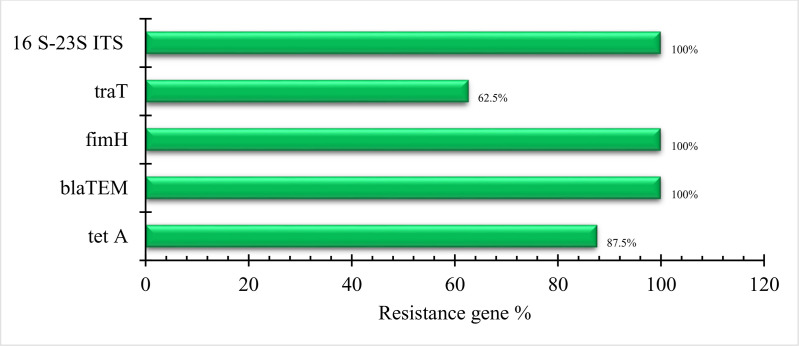
Prevalence (%) of virulence and antibiotic resistance genes. *K. pneumoniae* 16S-23S ITS (The 16S-23S rRNA gene internal transcribed spacer (ITS) regions of *Klebsiella*); traT (outer membrane protein-coding gene); fimH (the adhesive subunit of type 1 fimbriae); ESBL-encoding genes: bla_TEM_ (common extended-spectrum β-lactamase genes); Tetracycline resistance gene: *tetA* (efflux-mediated tetracycline resistance).

**Fig 4:**
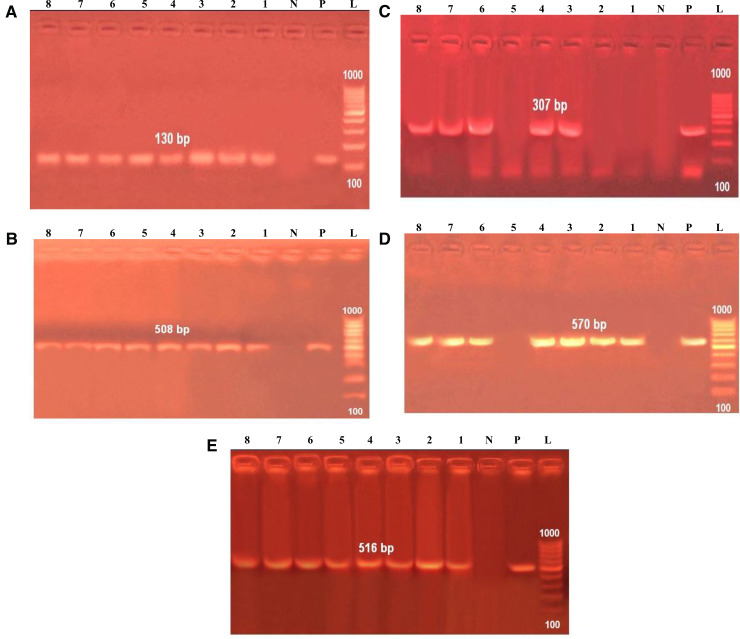
Agarose gel electrophoresis of PCR amplification products. **(A)**: Agarose gel electrophoresis of amplification products of 16S-23S ITS gene for characterization of K. pneumonia. Lane L: Molecular size marker (100-1000 bp). Lane P. and **N.**: Positive and negative controls. Lane1-8: Positive for K. pneumoniae strains at amplicon of 130 bp. **(B)**: Agarose gel electrophoresis of PCR amplification products of fimH gene for characterization of K. pneumonia. Lane L: Molecular size marker (100-1000 bp). Lane P. and **N.**: Positive and negative controls. Lane1-8: Positive for K. pneumoniae strains at amplicon of 508 bp. **(C)**: Agarose gel electrophoresis of PCR amplification products of TraT gene for characterization of K. pneumonia. Lane L: Molecular size marker (100-1000 bp). Lane P. and **N.**: Positive and negative controls. Lane 3,4,6,7 and 8: Positive for K. pneumoniae strains at amplicon of 307 bp. **(D)**: Agarose gel electrophoresis of PCR amplification products of tetA(A) gene for characterization of K. pneumonia. Lane L: Molecular size marker (100-1000 bp). Lane P. and **N.**: Positive and negative controls. Lane 1,2,3,4,6,7and 8: Positive for K. pneumoniae strains at amplicon of 570 bp. **(E)**: Agarose gel electrophoresis of PCR amplification products of blaTEM gene for characterization of K. pneumonia. Lane L: Molecular size marker (100-1000 bp). Lane P. and **N.**: Positive and negative controls. Lane 1- 8: Positive for K. pneumoniae strains at amplicon of 516 bp.

Among 90 pooled samples (organs and cloacal swabs) collected from eight commercial broiler farms, eight isolates (8.9%) were confirmed as *Klebsiella pneumoniae* through microbiological and molecular analysis. PCR amplification using primers and conditions specified in Table 1 successfully detected target sequences in these isolates. Subsequent characterization revealed distinct patterns of virulence and antibiotic resistance genes, as documented in Table 2, and Fig 3. Fig 4 presents the electrophoretic separation of PCR products, demonstrating the genetic profiles of antibiotic resistance determinants in the *K. pneumoniae* isolates.

**Table 1:** Target genes, Primers sequences, Amplicon sizes, and Amplification cycling conditions.

Target genes	Primers sequences	Amplicon sizes (bp)	Primary denaturation	Amplification (35 cycles)			Final extension	Reference
				Secondary denaturation	Annealing	Extension		
***Tet(A)***	GGTTCACTCGAACGACGTCA	570	94˚C 5 min.	94˚C 30 sec.	50˚C 40 sec.	72˚C 45 sec.	72˚C 10 min.	Randall *et al.* 2004
	CTGTCCGACAAGTTGCATGA							
***blaTEM***	ATCAGCAATAAACCAGC	516	94˚C 5 min.	94˚C 30 sec.	54˚C 40 sec.	72˚C 45 sec.	72˚C 10 min.	Colom *et al*., 2003
	CCCCGAAGAACGTTTTC							
***fimH***	TGCAGAACGGATAAGCCGTGG	508	94˚C 5 min.	94˚C 30 sec.	50˚C 40 sec.	72˚C 45 sec.	72˚C 10 min.	Ghanbarpour and Salehi, 2010
	GCAGTCACCTGCCCTCCGGTA							
***TraT***	GATGGCTGAACCGTGGTTATG	307	94˚C 5 min.	94˚C 30 sec.	55˚C 40 sec.	72˚C 40 sec.	72˚C 10 min.	Kaipainen *et al.*, 2002
	CACACGGGTCTGGTATTTATGC							
***16S-23S ITS***	ATTTGAAGAGGTTGCAAACGAT	130	94˚C 5 min.	94˚C 30 sec	55˚C 30 sec	72˚C 30 sec.	72˚C 7 min.	Turton *et al*., 2010
	TTCACTCTGAAGTTTTCTTGTGTTC							

**Table 2:** Molecular detection of virulence and antibiotics resistance genes in *K. pneumoniae isolates.*

K. pneumoniae isolates	*tetA(A)*	*bla*_*TEM*_	*fimH*	*TraT*	*16 S-23S ITS*
KP1	+	+	+	–	+
KP2	+	+	+	–	+
KP3	+	+	+	+	+
KP4	+	+	+	+	+
KP5	–	+	+	–	+
KP6	+	+	+	+	+
KP7	+	+	+	+	+
KP8	+	+	+	+	+

Green: Detected, Yellow: Un-detected.

### Antimicrobial susceptibility of Klebsiella pneumoniae

**Fig 5:**
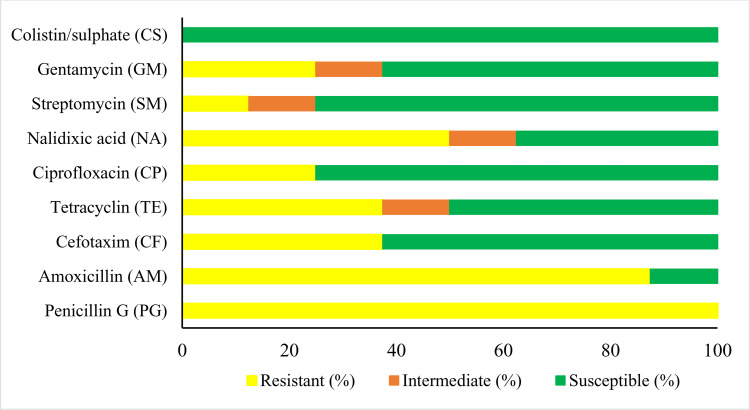
Antibiotic susceptibility test results %.

Regarding individual drugs, All *K. pneumoniae* isolates were resistant to Penicillin G (P) with percentage (100%) followed by Amoxicillin (AMX) with percentage (87.5%), Nalidixic acid (NA) with percentage (50%), Cefotaxime (CTX) and Tetracycline (TE) with percentage (37.5%). The *K. pneumonia* isolates were resistant to Gentamycin (CN), Ciprofloxacin (CIP) and Streptomycin(S) with percentage (25%), (25%) and (12.5%), respectively. In contrast, all isolates were sensitive to Colistin/sulphate (CTS) with percentage (100%), Table 3, and Fig 5.

**Table 3:** Antibiotic susceptibility test results of 8 isolated isolates.

Antibiotics	µg/disk	Resistant (%)	Intermediate (%)	Susceptible (%)
Penicillin G	**10**	100	0	0
Amoxicillin	**10**	87.5	0	12.5
Cefotaxime	**30**	37.5	0	62.5
Tetracycline	**30**	37.5	12.5	50
Ciprofloxacin	**5**	25	0	75
Nalidixic acid	**30**	50	12.5	37.5
Streptomycin	**10**	12.5	12.5	75
Gentamycin	**10**	25	12.5	62.5
Colistin/sulphate	**10**	0	0	100

Among 8 suspected *K. pneumoniae* isolates, 5 isolates with percentage (62.5%) are showed phenotypic multidrug resistant (MDR) against Penicillin G; Amoxicillin; Nalidixic acid; Cefotaxime and Tetracycline antibiotics. Also, the MAR (Multiple antibiotic resistance) index ranged from 0.22 to 0.88, Table 4.

**Table 4:** Antibiotic susceptibility profiles of K. pneumoniae isolates.

K.pneumoniae isolates	Antibiotic resistance pattern	MAR index*	Resistance pattern
KP1	PG,AM,NA	0.33	MDR**
KP2	PG,CF	0.22	–
KP3	PG,AM,NA	0.33	MDR
KP4	PG,AM,TE	0.33	MDR
KP5	PG,AM	0.22	–
KP6	PG,AM	0.22	–
KP7	PG,AM,CF,TE,CP,NA,CN	0.77	MDR
KP8	P,AM,CF,TE,CP,NA,SM,CN	0.88	MDR

*MAR Index (Multiple antibiotic resistance Index),**MDR: Multidrug resistance.

### Molecular identification and detection of some virulence and antibiotics resistance genes in *K. pneumoniae isolates*

PCR analysis of the *16*S-23*S ITS* region successfully identified all eight biochemically confirmed *Klebsiella pneumoniae* isolates, producing a distinct 130 bp amplicon (100% detection rate; Table 2, Fig 3). Virulence gene profiling revealed the *FimH* gene (508 bp; encoding type 1 fimbrial adhesin) in all isolates (100%), while the *TraT* gene (307 bp; encoding transfer protein T) was detected in 62.5% of isolates. Antibiotic resistance screening showed high prevalence of the *tetA(A)* gene (570 bp; 87.5% occurrence) conferring tetracycline resistance, and universal presence of the *blaTEM* gene (516 bp; 100%) indicating β-lactamase-mediated ampicillin resistance. These molecular characteristics are summarized in Table 2, Fig 3, and illustrated in the electrophoretic profiles of Fig 4.

### *In vivo* experiment

#### Clinical signs of birds infected with *Klebsiella pneumoniae*.

The infected birds exhibited lethargy, weakness, reduced appetite (anorexia), weight loss, dyspnea (difficulty breathing), whitish watery diarrhea, and lameness. These clinical signs were most severe in KT (untreated infection), while KPT and KAT showed a progressive reduction in symptom severity, likely due to treatment interventions, Fig 1.

#### Postmortem findings in birds infected with *Klebsiella pneumoniae.*

Postmortem examination of the infected birds revealed air sacculitis (inflammation of the air sacs), severe congestion of the liver and spleen, and renal congestion with ureteral urate deposition, Fig 2.

### *K. pneumoniae* re-isolation rate post challenge

The control group (CT), which remained uninfected and untreated, and PT group demonstrated complete absence of pathogen re-isolation throughout the study period. Similarly, both probiotic-treated (KPT) and antibiotic-treated (KAT) groups exhibited progressively declining re-isolation rates, with significant reductions observed by day 3 and day 7 post-infection, ultimately reaching zero by day 14. In contrast, the infected but untreated group (KT) maintained consistently higher pathogen recovery rates, with detectable levels persisting through days 1, 7, and 14 post-infection.

### Growth performance

Table 5 shows the Composition and chemical analysis of the basal experimental diets throughout the experiment. The broilers had comparable starting body weights across all experimental groups. Neither probioenzyme nor antibiotic supplementation had a significant effect on body weight or feed consumption during the first 10 days (*P > 0.05*; see Table 6). After *Klebsiella* infection, substantial growth retardation was detected on day 22 (*P < 0.05*), following the completion of antibiotic therapy. However, both the probioenzyme (KPT) and antibiotic (KAT) supplementation groups similarly showed significantly improved body weight (FBW) (*P < 0.05*), increased weight gain (WG), specific growth rate (SGR), feed conversion efficiency (FCE) (*P < 0.05*), and reduced feed conversion ratio (FCR) (*P < 0.05*). By day 35, the *Klebsiella*-challenged (KT) group exhibited decreased growth parameters and a reduction in cumulative feed intake. In contrast, both the probioenzyme (KPT) and antibiotic (KAT) groups-maintained feed intake at control (CT) levels, which corresponded with their continued, significant improvements in FBW, WG, SGR, and FCE (*P < 0.05*). Notably, the probioenzyme (KPT) restored FCR to a level statistically equivalent to the unchallenged control (CT) and antibiotic-treated groups (KAT). Moreover, probioenzyme non challenged group (PT) demonstrated the highest growth performance rate (*P < 0.05*).

**Table 5:** Composition and chemical analysis of the basal experimental diets*.

Ingredient Name	Starter	Grower	Finisher
	(0–13day) (Kg/1000)	(1426 day) (Kg/1000)	(27–35 day) (Kg/1000)
Corn, yellow grain	565	601	662.5
Soybean meal, 46%	366	326	245
Corn gluten meal, 60%	25.5	19	38
Limestone	17	16	16
Mono calcium phosphate	9.5	10	8
Sunflower oil	4.3	15	17
Nacl	3	3	3
Broiler Premix**	3	3	3
L-Lysine HCl, 98%	2	2.5	2.5
DL-methionine,99%	1.6	1.4	1.4
Sodium bicarbonate	1	1	1.5
Anti-mycotoxin	1	1	1
Anti-coccidia (diclazuril)	0.5	0.5	0.5
kemzyme	0.5	0.5	0.5
phytase	0.1	0.1	0.1
**Chemical analysis on DM basis:**			
Crude protein, %	23.16	21.2	19.11
Metabolizable energy, kcal	3000	3100	3200
Crude lipid, %	3	4.14	4.6
Crude fiber, %	2.75	2.7	2.5
Calcium, %	0.9	0.87	0.8
Available phosphorous, %	0.45	0.44	0.4
Chloride, %	0.22	0.22	0.21
Sodium, %	0.16	0.16	0.16
**Essential amino acids analysis**			
Lysine, %	1.44	1.28	1.16
Methionine, %	0.56	0.51	0.5
Methionine +cystine, %	0.95	0.87	0.81
Threonine, %	0.93	0.85	0.78
Arginine, %	1.52	1.3	1.2
Leucine, %	1.85	1.7	1.54
Isoleucine, %	1.18	1.05	0.97
Tryptophan, %	0.28	0.25	0.23
Valine, %	1.07	0.95	0.91
Phenyl alanine, %	1.12	1.04	0.97

*Formulated according to Avian nutrition specifications [105], and Chemical analysis was performed according to AOAC (AOAC 1994).

**Premix Supplied per kg of premix: *trans*-retinol(A), 12,500,000 IU; cholecalciferol(D3), 500,000 IU; α-tocopherol acetate(E), 75,000 mg; thiamine(B1), 4500 mg; riboflavin(B2), 8000 mg; pyridoxine(B6), 5000 mg; vitamin B12, 22,000 mg; pantothenic acid, 20,000 mg; folic acid, 2000 mg; biotin, 200,000 μg; Fe, 100,000 mg; Co,250 mg; Mn, 100 mg; Cu, 10,000 mg; Zn, 80,000 mg; I, 1000 mg; Se, 300 mg; Mo, 0.5 mg; Ca, 7.7%; P, 0.01%; Na, 0.18%; Ash, 97%.

**Table 6:** Growth performance of broilers chickens fed tested diets for 35 days. *.

Parameters	Test groups**				
	CT	KT	KAT	PT	KPT
**1-10 days**					
Final weight, (g/bird)	310 ± 10	315 ± 5	329 ± 6	348 ± 15	348 ± 5
Weight gain, (g/bird)	253 ± 10	258.15 ± 5.15	272.05 ± 5.95	291.1 ± 15	291.1 ± 5
Feed intake, (g/bird)	339.5 ± 4.5	343.5 ± 11.5	365.5 ± 10.5	367.5 ± 2.5	362.5 ± 3.5
Feed conversion ratio	1.345 ± 0.08	1.33 ± 0.02	1.34 ± 0.01	1.265 ± 0.08	1.245 ± 0.005
Specific growth rate	4.84 ± 0.1	4.89 ± 0.05	5.01 ± 0.05	5.17 ± 0.12	5.17 ± 0.04
Feed conversion efficiency	0.75 ± 0.04	0.75 ± 0.01	0.75 ± 0.005	0.8 ± 0.05	0.81 ± 0.005
**1-22 days**					
Final weight, (g/bird)	1210 ± 10^ab^	1130 ± 20^a^	1250 ± 10^bc^	1300 ± 20^c^	1225 ± 15^bc^
Weight gain, (g/bird)	1153 ± 10^ab^	1073.15 ± 19.85^a^	1193.05 ± 9.95^bc^	1243.1 ± 20^c^	1168.1 ± 15^bc^
Feed intake, (g/bird)	1606 ± 4	1545 ± 5	1565 ± 5	1615 ± 15	1580 ± 30
Feed conversion ratio	1.39 ± 0.01^ab^	1.44 ± 0.02^b^	1.315 ± 0.005^ab^	1.3 ± 0.01^a^	1.355 ± 0.045^ab^
Specific growth rate	8.73 ± 0.02^b^	8.54 ± 0.04^a^	8.83 ± 0.03^bc^	8.94 ± 0.04^c^	8.77 ± 0.04^bc^
Feed conversion efficiency	0.715 ± 0.005^ab^	0.695 ± 0.015^a^	0.765 ± 0.005^b^	0.765 ± 0.005^b^	0.74 ± 0.02 ^ab^
**1-35 days**					
Final weight, (g/bird)	2150 ± 10^b^	1865 ± 65^a^	2125 ± 25^b^	2291 ± 8.75^b^	2092 ± 52^b^
Weight gain, (g/bird)	2093 ± 10^b^	1808.15 ± 65^a^	2068.05 ± 25.1^b^	2234.35 ± 8.75^b^	2035.1 ± 52^b^
Feed intake, (g/bird)	3025 ± 25	2915 ± 65	3000 ± 50	3100 ± 50	2925 ± 25
Feed conversion ratio	1.445 ± 0.015^a^	1.61 ± 0.02^b^	1.45 ± 0.01^a^	1.385 ± 0.015^a^	1.44 ± 0.05^a^
Specific growth rate	10.38 ± 0.015 ^b^	9.97 ± 0.09^a^	10.34 ± 0.04^b^	10.56 ± 0.01^b^	10.3 ± 0.07^b^
Feed conversion efficiency	0.69 ± 0.01^ab^	0.62 ± 0.00^a^	0.69 ± 0.00^ab^	0.72 ± 0.01^b^	0.695 ± 0.25^ab^

* Values are means of five replicates(n = 5) ± S.E.M. Within a row, means with the same superscripts are not significantly different (*P* > 0.05). **CT: control fed on basal diets; KT: group fed on basal diets and challenged with *k. pneumonae;* KAT: group fed on basal diet, challenged with *k. pneumonae*, and treated with selected antibiotic; PT; group fed on basal diet enriched with probioenzyme (1g/kg diet); KPT: group fed on probioenzyme incorporated diet and challenged with *k. pneumonae*.

### Gut, lung, and liver histomorphology

**Fig 6:**
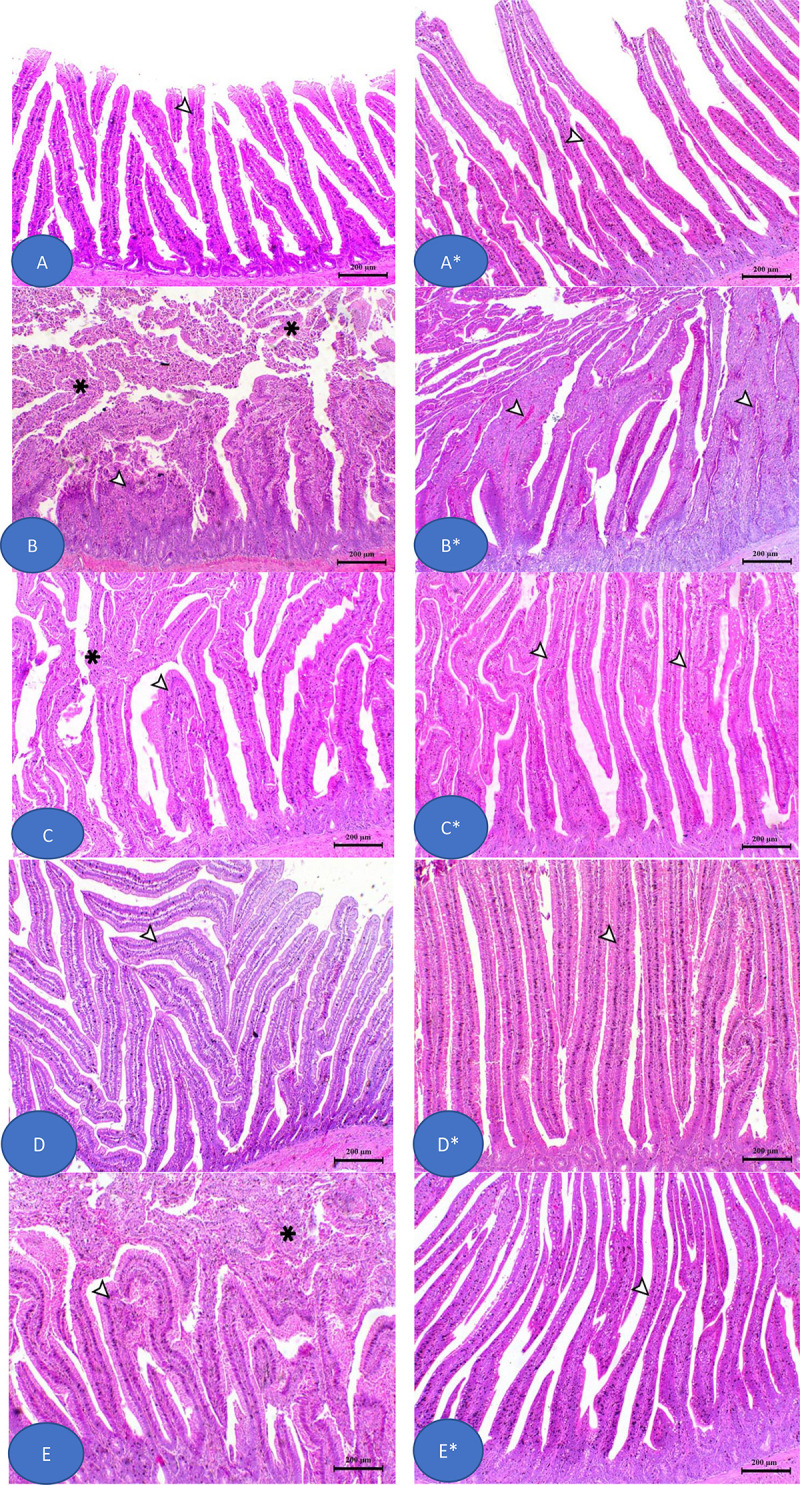
The histomorphology of broiler intestine after at 18 and 35-day feeding experiment. Stain H&E. Bar = 200 µm. At 18 Day point; **(A)** Intestine of control bird (CT); **(B)** Intestine of *k. pneumonaie* challenged group (KT); **(C)** Intestine of *k. pneumonaie* challenged and antibiotic treated group (KAT); **(D)** Intestine of probioenzyme supplemented group (PT); **(E)** Intestine of *k. pneumonaie* challenged and probioenzyme supplied group (KPT). At 35 Day point; (A*) Intestine of control bird (CT); (B*) Intestine of klebsiella group (KT); (C*) Intestine of KAT group; (D*) Intestine of probioenzyme group (PT); (E*) Intestine of KPT group.

**Fig 7:**
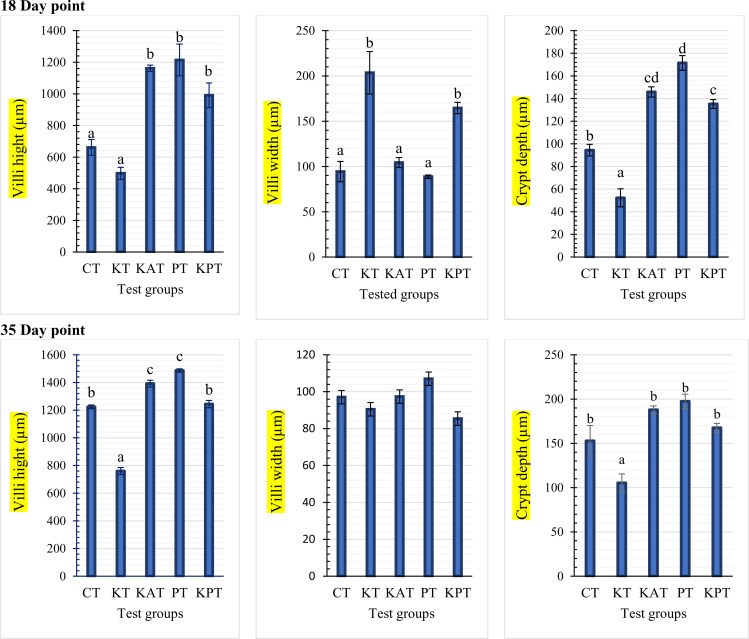
The intestinal morphometrical indices of broilers after 18 days. (A) villi height, (B) villi width, and (C) crypt depth) and 35-day feeding period (A*) villi height, (B*) villi width, and (C*) crypt depth). Bars with different letters differ statistically (*P < 0.05*).

Fig 6 illustrates the histopathological features of jejunum tissues from broiler chickens across experimental groups, including the control (CT), challenged (KT), probioenzyme non-challenged (PT), and groups fed KPT- and KAT-supplemented diets with subsequent *K. pneumoniae* infection. At day 18, the CT group maintained normal intestinal villi with intact pseudostratified squamous epithelium and goblet cells, while the KT group exhibited severe necrotic enteritis featuring mucosal epithelium loss, villous blunting and sloughing, persistent enteritis with villous adhesions, inflammatory cell infiltration, and necrotic cores that continued through day 35. Both KPT and KAT supplementation demonstrated protective effects against the *K. pneumoniae* challenge, significantly improving villus architecture and crypt depth compared to KT, particularly by trial completion. Supporting these observations, Fig 7 presents morphometric analysis (villus height, width, and crypt depth) after 18 and 35 days, revealing that KPT supplementation restored intestinal histology to near-CT levels, while PT showed the greatest morphological enhancement among all groups, and KT consistently displayed the poorest morphometric indices throughout the experimental period.

**Fig 8:**
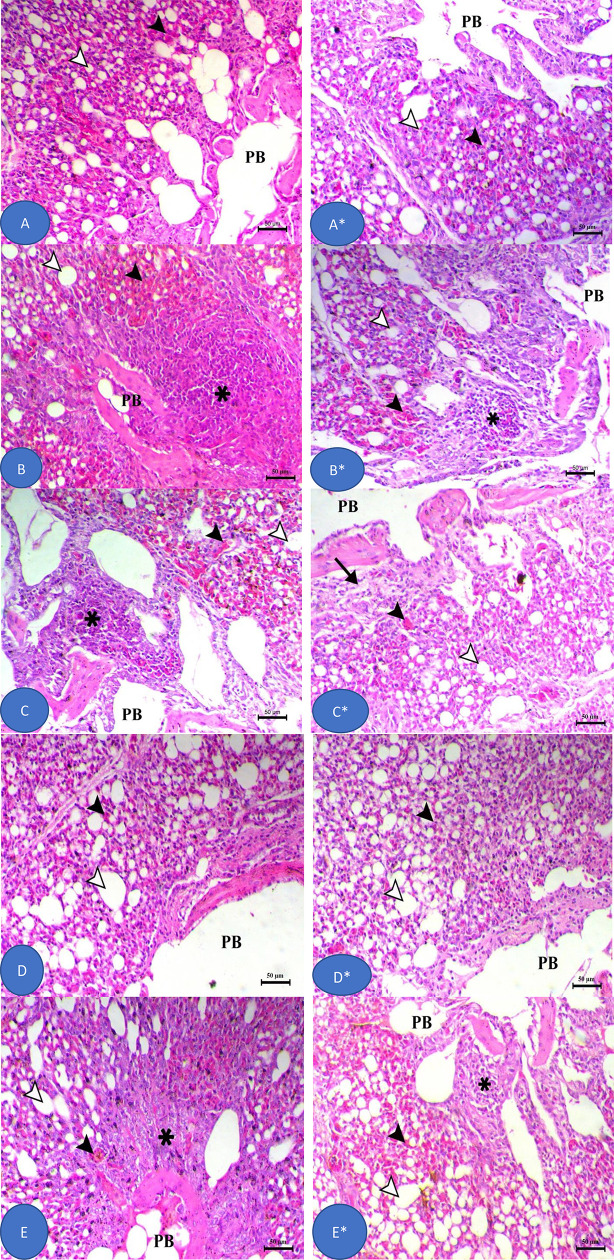
The histomorphology of broiler lung after 18 and 35-day feeding experiment. Stain H&E. Bar = 50 µm. At 18 Day point; **(A)** Lung of control bird (CT); (B) lung of *k. pneumonaie* challenged group (KT); (C) lung of *k. pneumonae* challenged and antibiotic treated group (KAT); (D) lung of probioenzyme supplemented group (PT); (E) lung of *k. pneumonae* challenged ant probioenzyme supplied group (KPT). At 35 Day point; (A*) Lung of control bird (CT); (B*) lung of klebsiella group (KT); (C*) lung of KAT group; (D*) lung of probioenzyme group (PT); (E*) lung of KPT group. PB: parabronchus.

Fig 8 revealed that at 18- and 35 days, the lungs of control birds (18 day) (CT) exhibited normal central parabronchi (PB) with intact air and blood capillaries. In contrast, lungs from the KT group at 18 days displayed peribronchial and interstitial pneumonia, characterized by alveolar capillary congestion, reduced air spaces, and significant mononuclear inflammatory cell infiltration. In contrast, by 35 days, lungs from the KT group still demonstrated features of interstitial pneumonia, including persistent alveolar capillary congestion, further decreased air spaces, and continued peribronchial mononuclear inflammatory cell infiltration. At day 18, Lung of KPT and KAT groups showed marked decrease in pneumonia features with decreased capillary congestion, increased air capillaries and focal peribronchial mononuclear cells infiltration. At day 35, the KPT group showed a significant decrease in pneumonia features, including decreased capillary congestion, increased air capillaries, and mild peribronchial fibrosis.

**Fig 9:**
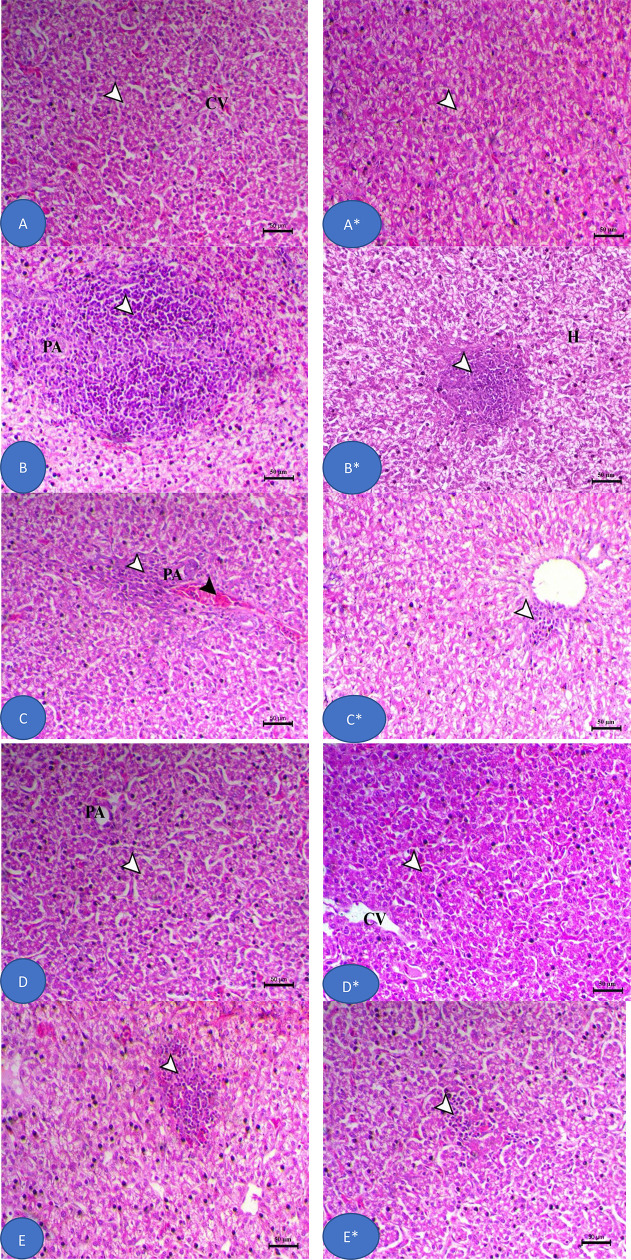
The histomorphology of broiler liver after at 18 and 35-day feeding experiment. Stain H&E. Bar = 50 µm. At 18 Day point; **(A)** Liver of control bird (CT); **(B)** Liver of *k. pneumonaie* challenged group (KT); **(C)** Liver of *k. pneumonaie* challenged and antibiotic treated group (KAT); **(D)** Liver of probioenzyme supplemented group (PT); **(E)** Liver of *k. pneumonaie* challenged and probioenzyme supplied group (KPT). At 35 Day point; (A*) Liver of control bird (CT); (B*) Liver of klebsiella group (KT); (C*) Liver of KAT group; (D*) Liver of probioenzyme group (PT); (E*) Liver of KPT group. CV: cytoplasmic vacuolation.PA: periportal.

Fig 9 revealed that at both sampling points, the liver tissue in the CK (control) and PT groups exhibited typical hepatocyte morphology with mild cytoplasmic vacuolation, indicative of glycogen accumulation, primarily around the central vein. In contrast, the KT group at 18 days displayed extensive lymphocytic infiltrations replacing hepatic tissue, along with periportal necrotic foci (PA) and significant mononuclear cell infiltration (predominantly lymphocytes); by 35 days, this progressed to diffuse hepatic vacuolation (H), necrotic foci, and marked infiltration of mononuclear cells (lymphocytes and macrophages). Meanwhile, the KPT & KAT groups at 18 days showed mild portal vein congestion and slight periportal mononuclear inflammatory cell infiltration (PA), which by 35 days persisted as mild hepatic vacuolation with only minimal periportal mononuclear inflammatory cell infiltration.

### Antioxidant and lysozyme activity

**Fig 10:**
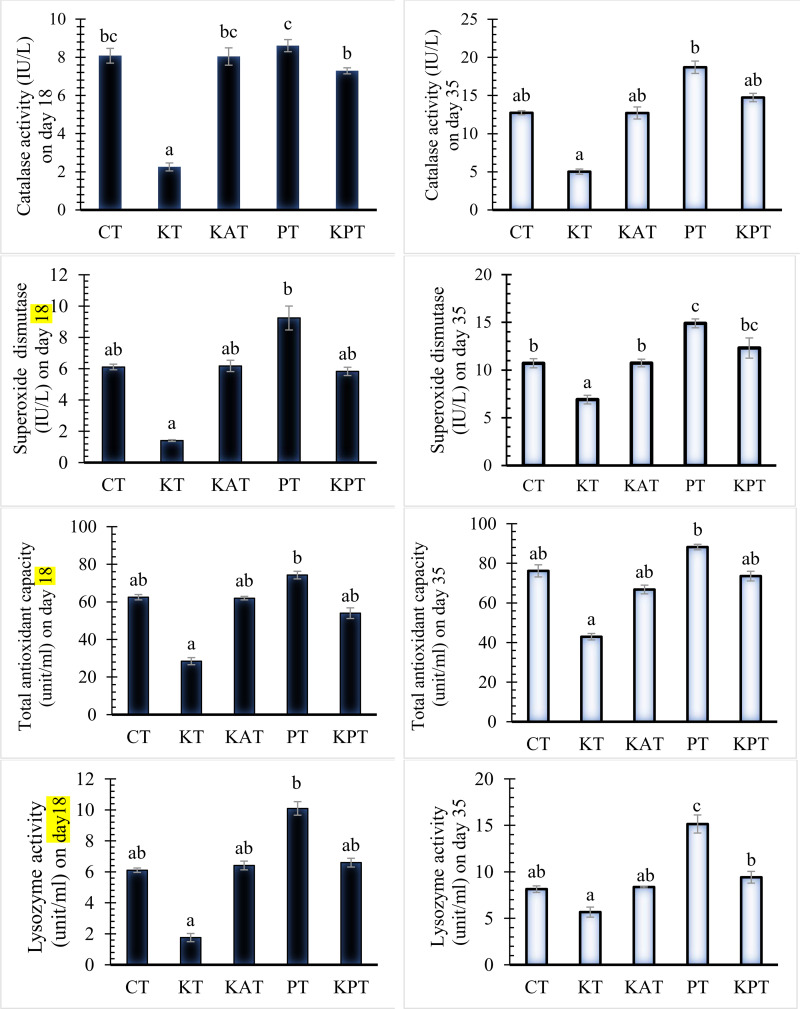
Antioxidant enzymes and lysozyme activity in broiler after 18 and 35-day feeding period. **(A)** Catalase activity (IU/L) on day 18; **(B)** Superoxide dismutase (IU/L) on day 18; **(C)** Total antioxidant capacity (unit/ml) on day 18; **(D)** Lysozyme activity (unit/ml) on day18. (A*) Catalase activity (IU/L) on day 35; (B*) Superoxide dismutase (IU/L) on day 35; (C*) Total antioxidant capacity (unit/ml) on day 35; (D*) Lysozyme activity (unit/ml) on day35. Bars with different letters differ statistically (*P < 0.05*).

Serum antioxidants and lysozyme activities are presented in Fig 10. From day 18 onward, all tested groups exhibited significant differences (*P* < 0.05). At both sampling points (days 18 and 35), the KT group showed the lowest antioxidant activity (TAC, CAT, and SOD) and lysozyme levels among all groups (*P* < 0.05), despite a slight increase by the end of the trial. In contrast, the PT group displayed the highest values for these markers.

At day 18, no significant differences (*P* > 0.05) were observed among the KPT, KAT, and CT groups in SOD, TAC, and lysozyme activity; however, KPT exhibited lower catalase (CAT) activity (*P* < 0.05). By day 35, KPT, KAT, and CT groups demonstrated comparable results, except for SOD and lysozyme activities, which were significantly higher in case of KPT group compared to CT and KAT groups, with only slight improvements in the measured parameters by the end of the trial.

## Discussion

The global rise in antimicrobial resistance (AMR) and the phasing out of antibiotic growth promoters (AGPs) in poultry production have intensified the search for sustainable alternatives. This study demonstrates that a novel probioenzyme formulation is a functionally effective alternative to therapeutic antibiotics, successfully mitigating a challenging multidrug-resistant (MDR) *Klebsiella pneumoniae* infection in broilers. The probioenzyme not only matched the antibiotic colistin in restoring growth and health but also provided superior enhancement of systemic antioxidant and immune responses [32,33].

*Klebsiella pneumoniae*, an opportunistic pathogen [34],represent a critical concern in poultry production due to its association with respiratory infections, mortality, and production losses, particularly in young chicks [35]. Our initial epidemiological survey underscores the seriousness of the challenge*. Klebsiella pneumoniae* was detected in 8.88% (8/90) of clinically diseased broiler chickens, aligning with prevalence rates reported by [36]. However, disparities exist across regions, while [37] and [38] reported similar rates in Indonesia and Egypt, respectively, [35] documented a markedly higher prevalence (67.2%) in Ethiopia. Such variations may reflect differences in farm management, antibiotic usage, or regional strain virulence.

Antimicrobial resistance (AMR) is an increasing global challenge, notably in poultry production, where antibiotics are commonly used to treat diseases and promote growth. The overuse of antibiotics in poultry farming has exacerbated antimicrobial resistance (AMR), with commensal bacteria such as *Klebsiella pneumoniae* evolving into multidrug-resistant (MDR) pathogens [39,40]. In this study, all isolates exhibited resistance to penicillin G (100%), consistent with findings by [41,42] and [43], This high resistance rate is likely due to penicillin G’s widespread use for flock therapy and growth promotion. Similarly, resistance result to amoxicillin (87.5%) mirrored results from [44–46]. Additionally,cefotaxime was shown 37.5% resistance to isolated *k. pneumonae* which is similar to [47] who reported 42%, but this result was lower than reported by [18] which reported 75.3% resistance.

Additionally, the emergence of fluoroquinolone resistance may be rising due to its consequent high-usage in the treatment of bacterial diseases [48]. In this study, resistance to nalidixic acid resistance (50%), which contrasted sharply with [38]; 94.6%), suggesting regional differences in fluoroquinolone usage. The resistance result to nalidixic acid (50%), which contrasted sharply with [38] who reported that 94.6% of the isolates were resistant to nalidixic acid.

Tetracycline resistance can occur through ribosomal protection, efflux pumps, or enzymatic modification. In this study, *Klebsiella pneumoniae* isolates showed resistance to tetracycline revealed (37.5%) similar to [49], who reported that *K. pneumoniae* isolated from chicken farms in Blitas district in Indonesia showed resistance to Tetracycline 35.72%, but this result diverged from higher rates reported by [50], 71% and [38], 83.8%. All isolates remained sensitive to colistin (100%), corroborating [51] who reported that all isolates exhibited (100%) sensitive to colistin and [50], who reported that Colistin-resistant *K. pneumoniae* rates were low (4%). Although resistance rates up to 18.9% have been observed by [38], underscoring the urgency of antimicrobial stewardship. This finding uncovers a dangerous upward trend in antibiotic resistance, signaling growing difficulties in treating infections with standard drugs.

Among the isolates, 62.5% (5/8) displayed MDR phenotypes, primarily against penicillin G, amoxicillin, nalidixic acid, cefotaxime, and tetracycline—a profile comparable to [50] (67%), in contrast to [52] and [38], who found that all isolates of *K. pneumoniae* were MDR. Furthermore, the multiple antibiotic resistance (MAR) index in this study ranged from 0.22 to 0.88, whereas [38] found that the MAR index ranged from 0.19 to 0.94 of all *K. pneumoniae* isolates, with the majority of chicken isolates having an index between 0.68 and 0.81, and [53] found that the index ranged from 0.071 to 1 among *K. pneumoniae* isolates from broilers. The K. pneumoniae-resistant isolates were screened for the presence of *bla*_TEM_, a gene encoding a β-lactamase enzyme that hydrolyzes β-lactam antibiotics, thereby conferring resistance to penicillin, amoxicillin, and cefotaxime [54]. These findings suggest that the presence of different β-lactamase genes (*bla*_TEM_ and potentially others) may contribute to varying levels of antibiotic resistance [55]. Furthermore, PCR detection based on *16*S-23*S ITS* internal transcribed spacer (ITS) of *K. pneumoniae* was carried out in the present study in which eight isolates of *K. pneumoniae* were positive for *16*S-23S *ITS* gene (Fig 3). This agreed with that reported by [56] and [57]. PCR analysis confirmed the presence of virulence associated genes (*fimH*: 100%, and *traT*: 62.5%) and resistance determinants (*tetA*: 87.5%, and *bla*_*TEM*_: 100%), consistent with global trends [58–60]. The *fimH* gene, critical for adhesion, was ubiquitous, while *traT*’s lower prevalence [60,61] suggests strain-specific pathogenicity.

The microbiological investigations confirmed the widespread distribution of *Klebsiella pneumoniae* throughout poultry production systems. The bacterium was consistently isolated from cloacal swabs, indicating active colonization of chickens’ gastrointestinal tracts. Environmental surveillance further detected its presence in farm feces, contaminated drinking water sources, and even in fecal samples collected from veterinary staff and farm workers, demonstrating multiple potential transmission routes within these ecosystems [62,63]. The re-isolation rate of k. pneumonae in this study was decreased through using both probioenzyme and/or antibiotic.The reduced re-isolation rate of *K. pneumoniae* in flocks treated with colistin and probioenzyme highlights two key strategies,restrained deployment of last-line antibiotics such as colistin [18] and probiotic supplementation to competitively exclude pathogens [64]. Probiotics may act via bacteriocin production, adhesion blockade, or immune modulation [65], offering a viable alternative to AGPs while mitigating AMR risks [66]. From the point of public health, detection of *K. pneumoniae* in cloacal swabs underscores its zoonotic potential, with transmission risks via contaminated poultry products or farm environments [66,67]. Despite being considered low-pathogenicity, its MDR traits complicate treatment in immunocompromised hosts [68]. Moving forward, integrated approaches—combining probiotics, stringent biosecurity, and antibiotic stewardship—are essential to curb AMR and safeguard both animal and human health.

In our study, infected birds exhibited clinical signs including depression, decreased appetite, body weight loss, gasping, whitish watery diarrhea, and occasional lameness. These symptoms were most severe in KT Group (untreated challenged birds), while showing progressively reduced severity in KPT and KAT group, respectively. Similar result was demonstrated by [69,70]. Postmortem examination of K. pneumoniae-infected broilers revealed characteristic lesions including air sacculitis, severe hepatic and splenic congestion, and renal congestion with ureteral urate deposition. These findings nearly similar to that observed by [57]. Furthermore*, Klebsiella pneumoniae* infection significantly impaired growth performance in broilers, consistent with its pathogenic disruption of metabolic pathways and vital organ function. The observed growth suppression aligns with the bacterium’s capacity to induce systemic inflammation, gut barrier dysfunction, and metabolic disturbances [18].

Probioenzyme supplementation (KPT) restored final body weight and growth metrics to levels statistically indistinguishable from the antibiotic-treated (KAT) and unchallenged control (CT) groups, while the unsupplemented challenged group (KT) remained significantly lower. The improved growth performance is primarily attributed to the *Bacillus*-derived enzymes (protease, amylase) and phytase, which enhanced the availability of protein, carbohydrates, and phosphorus. Concurrently, the prebiotics (MOS, beta-glucan) supported probiotic colonization and directly stimulated gut-associated immune tissue. This contributed to the observed pathogen exclusion and elevated lysozyme activity, which further improved macronutrient digestion and gut health. These effects are reflected in the elevated villus height and crypt depth, indicating greater absorptive capacity. Finally, the hepatoprotective and antioxidant effects are likely linked to metabolites from the *Lactobacillus* and *Bifidobacterium* strains, as well as systemic reductions in inflammation stemming from a healthier gut [71–75].

Antibiotics directly suppress *K. pneumoniae* proliferation, alleviating inflammation-mediated appetite suppression and toxin-induced epithelial damage [76,77]. The severe necrotic enteritis and villous blunting observed in challenged, untreated (KT) birds illustrate the direct damage *K. pneumoniae* inflicts on the intestinal epithelium, compromising barrier function and nutrient absorption [78]. Infection triggers a robust inflammatory response characterized by elevated pro-inflammatory cytokine production and oxidative stress, ultimately leading to tissue damage in the gastrointestinal tract, impaired growth performance, and increased mortality rates in poultry flocks [79,80]. To counteract these detrimental effects, alternative approaches to antibiotics have demonstrated efficacy, including probiotic supplementation, prebiotic administration, and probioenzyme formulations (combination therapies) [81,82]. Both the probioenzyme (KPT) and antibiotic (KAT) interventions provided significant histopathological protection, restoring villus architecture and crypt depth. Probiotics within the formulation likely contributed through competitive exclusion of pathogens, stimulation of mucin production, and upregulation of tight junction proteins [12,83]. Concurrently, the included enzymes (e.g., phytase, proteases, xylanase) would have enhanced nutrient digestibility and availability, directly supporting the metabolic demands for recovery and growth [84,85]. This synergistic action on the gut likely explains the superior growth trends in the KPT group by trial end.

Key metrics for evaluating intestinal absorptive capacity include,Villus height (positively correlated with nutrient absorption efficiency), and Crypt depth (indicative of epithelial regenerative capacity) [86]. It is important to note that the observed benefits of the probioenzyme formulation are due to a synergistic or additive effect, as the control diet already contained commercial enzymes (kemzyme and phytase). The specific probiotic strains and prebiotics likely enhanced the activity of both natural and supplemented enzymes by modulating the gut environment, while also directly contributing to pathogen exclusion and immune modulation. This demonstrates the advantage of multi-component additives that target multiple physiological pathways. Moreover, Antibiotic administration leads to reduced villus-damaging bacterial toxins [18]. Probiotics exert protective effects through multiple pathways by Stimulation of mucin production, Upregulation of tight junction proteins, and Competitive exclusion of pathogenic bacteria [87].

The pathogenic effects of *K. pneumoniae* extended beyond the gastrointestinal tract, causing significant interstitial pneumonia and hepatic lesions featuring lymphocytic infiltration and necrosis. This bacterial invasion triggers a robust inflammatory response characterized by elevated pro-inflammatory cytokine production and oxidative stress, ultimately leading to gastrointestinal tissue damage, impaired growth performance, and increased mortality in poultry [78]. The probioenzyme’s efficacy in markedly reducing pulmonary inflammation and hepatic damage underscores its systemic protective benefits. These hepatoprotective and pulmonary protective effects align with literature on the ability of probiotic-enzyme combinations to modulate inflammation and oxidative stress in peripheral organs [88]. Both probiotic and antibiotic interventions improve these parameters, through distinct mechanisms – probiotics enhance epithelial development and tissue morphology, while antibiotics primarily reduce villus-damaging bacterial toxins [10]. The protective action of probiotics involves multiple pathways, such as stimulating mucin production, upregulating tight junction proteins, and competitively excluding pathogens [89], which collectively enhance gut health and performance. A limitation of this study is the lack of direct nutrient digestibility measurements. However, the significant improvements in feed conversion ratio (FCR) and villus morphology strongly indicate enhanced nutrient utilization. Future studies employing ileal digestibility assays are needed to conclusively attribute these effects to the enzymatic components. In this context, the growth and efficiency parameters serve as robust, integrated proxies for overall nutritional enhancement.

*Klebsiella pneumoniae* is an opportunistic bacterial pathogen that causes respiratory infections in poultry, leading to significant production losses and increased mortality rates [35]. Lung histopathology in infected chickens revealed severe diffuse interstitial pneumonia, exhibiting pronounced mononuclear cell infiltration and marked bronchial epithelial hyperplasia. This come in agree with previous studies [52,90]. Furthermore, Liver sections from infected chickens displayed moderate vacuolar degeneration of hepatocytes accompanied by scattered foci of coagulative necrosis. Probioenzyme administration significantly improved pulmonary and hepatic tissue architecture, as evidenced by histopathological analysis. These results align with established literature on the hepatoprotective and pulmonary protective effects of probiotic-enzyme combinations [91–93].

Oxidative stress poses a significant risk to livestock health as it generates excessive reactive oxygen species (ROS), overwhelming the antioxidant defense system and impairing its function [80]. Systemically, the infection induced significant oxidative stress, as evidenced by the dramatic suppression of serum antioxidant capacity (TAC, SOD, CAT) in the KT group. The probioenzyme not only normalized these parameters but, by day 35, elevated key antioxidants (SOD) and lysozyme activity above levels in both the control and antibiotic-treated groups. This suggests the formulation does more than just mitigate infection; it actively enhances the host’s innate immune and antioxidant defense systems [94–96]. The KPT group exhibited the highest TAC and SOD levels, while CAT activity remained consistent across the Control, KPT, and KAT groups. These results confirm that antibiotics exert their therapeutic effects by modifying bacterial metabolism, leading to bacteriocidal outcomes and/or reducing infection-mediated organ damage. This mechanism ultimately contributes to improved oxidative balance in the host [97]. In contrast, the probioenzyme treatment not only enhances antioxidant activity but also offers an environmentally sustainable alternative, reducing reliance on antibiotic-based growth promoters [98,99].

As a leucocyte-derived mucolytic enzyme, lysozyme constitutes a fundamental innate immune defense mechanism that actively protects against microbial invasion [100]. Lysozyme activity was markedly reduced in the KT-challenged birds but increased significantly in the KPT, CK, and KAT groups. In agreement with previous research, our result shows significantly higher serum lysozyme activity in birds supplemented with probioenzyme [101]. This probioenzyme formulation can enhance poultry immunity by boosting humoral defenses (including lysozyme), potentially improving broiler flock health and biosecurity. This immunomodulatory effect represents a critical strategy for decreasing antibiotic dependence in poultry production. Specifically, probiotics contribute to immune homeostasis, offering a sustainable alternative to conventional antimicrobial use [102,103]. In contrast, the antibiotic’s primary action is the direct bactericidal reduction of the pathogenic load, which indirectly alleviates infection-driven oxidative stress [104]*.*

This study’s significance lies in the direct, evidence-based comparison of a multi-component natural feed additive against a critical last-resort antibiotic for a defined MDR pathogen. We move beyond reporting general probiotic benefits to demonstrating that a specific commercial probioenzyme can match therapeutic antibiotic efficacy in a real-world disease challenge scenario, while concurrently boosting the host’s endogenous defense systems. This positions probioenzymes not merely as growth promoters but as viable tools for veterinary therapeutic or prophylactic support within antibiotic stewardship programs.

This study confirms the formulation’s efficacy, paving the way for future work on optimal dosing, long-term ecological impacts, and economic viability. A critical next step is to employ direct microbiome analysis to define the mechanistic basis, while also deconstructing the synergistic contributions of individual components to guide refined formulation.

## Conclusion

In summary, facing the persistent threat of MDR *K. pneumoniae* in poultry, dietary supplementation with the tested probioenzyme offers a powerful, sustainable alternative to antibiotics. It effectively controls infection, restores growth performance, protects vital organ integrity, and enhances systemic antioxidant and immune competence. Integrating such strategies into poultry health management is a crucial step toward reducing reliance on antibiotics, mitigating AMR risks, and achieving sustainable poultry production.
